# Cannabis use as a factor of lower corpulence in hepatitis C-infected patients: results from the ANRS CO22 Hepather cohort

**DOI:** 10.1186/s42238-022-00138-9

**Published:** 2022-06-11

**Authors:** Tangui Barré, Fabrice Carrat, Clémence Ramier, Hélène Fontaine, Vincent Di Beo, Morgane Bureau, Céline Dorival, Dominique Larrey, Elisabeth Delarocque-Astagneau, Philippe Mathurin, Fabienne Marcellin, Ventzislava Petrov-Sanchez, Carole Cagnot, Patrizia Carrieri, Stanislas Pol, Camelia Protopopescu, the ANRS/AFEF Hepather study group, Laurent Alric, Chloe Pomes, Fabien Zoulim, Marianne Maynard, Roxane Bai, Lucie Hucault, François Bailly, François Raffi, Eric Billaud, David Boutoille, Maeva Lefebvre, Elisabeth André-Garnier, Paul Cales, Isabelle Hubert, Adrien Lannes, Françoise Lunel, Jérôme Boursier, Tarik Asselah, Nathalie Boyer, Nathalie Giuily, Corinne Castelnau, Giovanna Scoazec, Stanislas Pol, Hélène Fontaine, Emilie Rousseaud, Anaïs Vallet-Pichard, Philippe Sogni, Victor de Ledinghen, Juliette Foucher, Jean-Baptiste Hiriart, Jancell M’Bouyou, Marie Irlès-Depé, Marc Bourlière, Si Nafa Si Ahmed, Valérie Oules, Albert Tran, Rodolphe Anty, Eve Gelsi, Régine Truchi, Dominique Thabut, Saloua Hammeche, Joseph Moussali, Xavier Causse, Barbara De Dieuleveult, Brahim Ouarani, Damien Labarrière, Nathalie Ganne, Véronique Grando-Lemaire, Pierre Nahon, Séverine Brulé, Betul Ulker, Dominique Guyader, Caroline Jezequel, Audrey Brener, Anne Laligant, Aline Rabot, Isabelle Renard, François Habersetzer, Thomas F. Baumert, Michel Doffoel, Catherine Mutter, Pauline Simo-Noumbissie, Esma Razi, Jean-Pierre Bronowicki, Hélène Barraud, Mouni Bensenane, Abdelbasset Nani, Sarah Hassani-Nani, Marie-Albertine Bernard, Georges-Philippe Pageaux, Dominique Larrey, Magda Meszaros, Sophie Metivier, Christophe Bureau, Thibault Morales, Jean Marie Peron, Marie Angèle Robic, Thomas Decaens, Marine Faure, Bruno Froissart, Marie-Noelle Hilleret, Jean-Pierre Zarski, Ghassan Riachi, Odile Goria, Fatima Paris, Hélène Montialoux, Vincent Leroy, Giuliana Amaddeo, Anne Varaut, Mélanie Simoes, Rachida Amzal, Olivier Chazouillières, Tony Andreani, Bénédicte Angoulevant, Azeline Chevance, Lawrence Serfaty, Didier Samuel, Teresa Antonini, Audrey Coilly, Jean-Charles Duclos-Vallée, Mariagrazia Tateo, Armand Abergel, Maud Reymond, Chanteranne Brigitte, Buchard Benjamin, Léon Muti, Claire Geist, Guillaume Conroy, Raphaëlle Riffault, Isabelle Rosa, Camille Barrault, Laurent Costes, Hervé Hagège, Véronique Loustaud-Ratti, Paul Carrier, Maryline Debette-Gratien, Philippe Mathurin, Guillaume Lassailly, Elise Lemaitre, Valérie Canva, Sébastien Dharancy, Alexandre Louvet, Anne Minello, Marianne Latournerie, Marc Bardou, Thomas Mouillot, Louis D’Alteroche, Didier Barbereau, Charlotte Nicolas, Laure Elkrief, Anaïs Jaillais, Jérôme Gournay, Caroline Chevalier, Isabelle Archambeaud, Sarah Habes, Isabelle Portal, Moana Gelu-Simeon, Eric Saillard, Marie-Josée Lafrance, Lucie Catherine, Fabrice Carrat, Frederic Chau, Céline Dorival, Isabelle Goderel, Clovis Lusivika-Nzinga, Marc-Antoine Bellance, Jonathan Bellet, Priscilla Monfalet, Jessica Chane-Teng, Sephora Bijaoui, Grégory Pannetier, François Téoulé, Jérôme Nicol, Florian Sebal, Rafika Bekhti, Carole Cagnot, Anaïs Boston, Laura Nailler, Guillaume Le Meut, Alpha Diallo, Ventzislava Petrov-Sanchez, Marc Bourlière, Jérôme Boursier, Fabrice Carrat, Patrizia Carrieri, Elisabeth Delarocque-Astagneau, Victor De Ledinghen, Céline Dorival, Hélène Fontaine, Slim Fourati, Chantal Housset, Dominique Larrey, Pierre Nahon, Georges-Philippe Pageaux, Ventzislava Petrov-Sanchez, Stanislas Pol, Mathias Bruyand, Linda Wittkop, Fabien Zoulim, Jessica Zucman-Rossi, Marianne L’hennaff, Michèle Sizorn, Carole Cagnot

**Affiliations:** 1 Aix Marseille Univ, Inserm, IRD, SESSTIM, Sciences Economiques & Sociales de la Santé & Traitement de l’Information Médicale, ISSPAM, Marseille, France; 2 Institut National de la Santé et de la Recherche Médicale (INSERM), Institut Pierre Louis d’Epidémiologie et de Santé Publique, Sorbonne Université, Paris, France; 3 Hôpital Saint-Antoine, Unité de Santé Publique, Assistance Publique-Hôpitaux de Paris (AP-HP), Paris, France; 4 Département d’Hépatologie/Addictologie, Université de Paris, AP-HP, Hôpital Cochin, Paris, France; 5 Liver Unit-IRB-INSERM 1183, Hôpital Saint Eloi, Montpellier, France; 6 Université Paris-Saclay, UVSQ, Inserm, CESP, Team Anti-infective Evasion and Pharmacoepidemiology, 78180 Montigny, France; 7 Epidemiology and Public Health Department, AP-HP, GHU Paris Saclay University, Raymond Poincaré Hospital, 92380 Garches, France; 8 Service des Maladies de l’Appareil Digestif, Université Lille 2 and Inserm U795, Lille, France; 9 ANRS|Emerging Infectious Diseases, Department of Clinical Research, Paris, France

**Keywords:** Cannabis, Marijuana, Hepatitis C, Chronic, Obesity, Body weight, Fibrosis, Behaviors, Δ9-Tetrahydrocannabinol, Corpulence, Endocannabinoid system

## Abstract

**Background:**

Patients with chronic hepatitis C virus (HCV) infection are at greater risk of developing metabolic disorders. Obesity is a major risk factor for these disorders, and therefore, managing body weight is crucial. Cannabis use, which is common in these patients, has been associated with lower corpulence in various populations. However, this relationship has not yet been studied in persons with chronic HCV infection.

**Methods:**

Using baseline data from the French ANRS CO22 Hepather cohort, we used binary logistic and multinomial logistic regression models to test for an inverse relationship between cannabis use (former/current) and (i) central obesity (i.e., large waist circumference) and (ii) overweight and obesity (i.e., elevated body mass index (BMI)) in patients from the cohort who had chronic HCV infection. We also tested for relationships between cannabis use and both waist circumference and BMI as continuous variables, using linear regression models.

**Results:**

Among the 6348 participants in the study population, 55% had central obesity, 13.7% had obesity according to their BMI, and 12.4% were current cannabis users. After multivariable adjustment, current cannabis use was associated with lower risk of central obesity (adjusted odds ratio, aOR [95% confidence interval, CI]: 0.45 [0.37–0.55]), BMI-based obesity (adjusted relative risk ratio (aRRR) [95% CI]: 0.27 [0.19–0.39]), and overweight (aRRR [95% CI]: 0.47 [0.38–0.59]). This was also true for former use, but to a lesser extent. Former and current cannabis use were inversely associated with waist circumference and BMI.

**Conclusions:**

We found that former and, to a greater extent, current cannabis use were consistently associated with smaller waist circumference, lower BMI, and lower risks of overweight, obesity, and central obesity in patients with chronic HCV infection. Longitudinal studies are needed to confirm these relationships and to assess the effect of cannabis use on corpulence and liver outcomes after HCV cure.

**Trial registration:**

ClinicalTrials.gov identifier: NCT01953458.

## Introduction

Patients with chronic hepatitis C virus (HCV) infection are at greater risk of metabolic disorders such as hyperlipidemia, hepatic steatosis, insulin resistance, metabolic syndrome, and diabetes mellitus (Chaudhari et al. 2021). Direct acting antivirals (DAA) can now cure HCV, leading to liver fibrosis regression, less liver necroinflammation, a lower risk of hepatic decompensation, amelioration of glycemic control, and lower liver-related and overall mortality (Kang et al. 2021; Roche et al. 2018; Huang et al. 2021; Cacciola et al. 2021). However, for patients with diabetes and/or cirrhosis, the benefits in terms of glycemic control and the lower risks of hepatocellular carcinoma (HCC) and mortality are not as strong (Cacciola et al. 2021; Váncsa et al. 2021; Benhammou et al. 2021). Moreover, obesity and hepatic steatosis are risk factors for HCC development after HCV cure (Minami et al. 2021; Ji et al. 2021).

Therefore, for patients with chronic HCV infection, managing metabolic disorders is crucial, both before and after HCV cure. In this population, elevated body weight and/or obesity are associated with hepatic steatosis (Younossi et al. 2004), insulin resistance (Delgado-Borrego et al. 2011), and type 2 diabetes mellitus (Wang et al. 2007). Accordingly, body weight management would appear to be a strong lever to prevent the development of metabolic disorders.

Studies in the general population have shown that cannabis use is inversely associated with body weight, body mass index (BMI), and the risk of both obesity and weight gain (Meier et al. 2019; Alshaarawy and Anthony 2019; Sidney 2016; Ngueta et al. 2015; Clark et al. 2018). However, apart from data on oral cannabinoid-containing medications against interferon and ribavirin-induced weight loss (Costiniuk et al. 2008), no data on the impact of real-life cannabis use on body weight in people with chronic HCV infection have been published. As HCV interacts with host lipid metabolism through several mechanisms (Serfaty 2017), and given that HCV infection is associated with higher levels of plasma endocannabinoids (Patsenker et al. 2015), the relationship between cannabis use and body weight may differ between patients with chronic HCV infection and the general population.

Using data from the French ANRS CO22 Hepather cohort, we aimed to identify clinical and socio-behavioral (including cannabis use) risk factors for obesity and overweight in people with chronic HCV-infection, a population where cannabis use prevalence is high (Barré et al. 2020).

## Material and methods

### Design and participants

ANRS CO22 Hepather is an ongoing French, national, multicenter, prospective cohort study of patients with chronic active or inactive HCV or HBV infection, which started in August 2012 (Pol et al. 2017). Eligible patients were invited to participate in the cohort during a medical visit in their hepatitis healthcare center. Thirty-two expert centers are involved throughout France. Sociodemographic, clinical, and biological data were collected at the enrolment visit. Patients are followed-up on a yearly basis, and supplemental data are collected during visits related to particular events (e.g., HCV or HBV therapy initiation). Written informed consent was obtained from each cohort participant before enrolment. The Hepather protocol was written in accordance with the Declaration of Helsinki and French law for biomedical research. It was approved by the “Comité de Protection des Personnes (CPP) Ile de France 3” Ethics Committee (Paris, France) and the French Regulatory Authority (ANSM).

### Study population

The present study population comprised patients with chronic HCV infection (defined as positive HCV-RNA and anti-HCV antibodies) at cohort enrolment. HCV-cured patients were therefore not included. Cohort exclusion criteria were HIV co-infection, receiving HCV treatment, and having stopped HCV treatment for less than 3 months at enrolment. For the present study, we also excluded patients co-infected with hepatitis B, those with no data for cannabis use, and patients with unavailable data for either waist circumference or BMI.

### Data collection

At the cohort enrolment visit, patients completed a face-to-face interview with their physician based on a structured questionnaire. Anthropological measurements and urine and blood samples were also taken.

The questionnaire collected clinical and sociodemographic data including sex, age, country of birth, educational level, average monthly household income, employment status (employed or not), time since HCV diagnosis, HCV treatment status, lifetime and current cannabis use, tobacco use, current and past alcohol consumption (number of standard drinks per day), and current coffee consumption (number of cups per day). Body height, weight, and waist circumference were measured. Data derived from blood samples included platelet count (10^9^/L), and aspartate aminotransferase (AST, IU/L) and alanine aminotransferase (ALT, IU/L) levels.

### Outcomes

There were two study outcomes, assessed according to waist circumference and BMI. The first was “central” obesity, defined as having a waist circumference ≥ 94 cm for men (except for men born in Asia, and Central or South America, for whom the cut-off was set at 90 cm) and ≥ 80 cm for women (Grundy et al. 2005). The second outcome was a three-category BMI-status variable. Participants with obesity (defined as a BMI ≥ 30 kg/m^2^), and those with overweight (defined as a BMI between 25 and 30 kg/m^2^), were compared with participants without obesity or overweight (World Health Organization 2019).

### Explanatory variables

In terms of cannabis use, people who answered “yes” to the question concerning current use were classified in the “current use” category. Among people who answered “no,” those who reported “yes” to the subsequent lifetime cannabis use question were classified in the “former use” category. Finally, people who answered “no” to both questions (current/lifetime use) were classified into the “never” category. Similarly, tobacco use was divided into “current use,” “former use,” and “never.” Coffee consumption was defined as none (0 cups per day), moderate (1–2 cups per day), or elevated (≥ 3 cups per day). The 3 cups/day threshold was chosen based on previous results showing a potential protective effect of coffee consumption on liver stiffness and mortality in patients likely to develop liver disease (Carrieri et al. 2018; 2017a; Protopopescu et al. 2018). Alcohol consumption was classified into the following three categories based on the threshold for unhealthy alcohol use (defined as > 2 and > 3 standard drinks per day for women and men, respectively, in accordance with the French National Authority for Health (Haute Autorité de Santé (French National Authority for Health) 2014)): (i) abstinent with no history of unhealthy use, (ii) current moderate alcohol use (i.e*.*, non-abstinent and non-unhealthy use), and (iii) unhealthy alcohol use (past or current).

As self-reported ethnicity was not collected in the cohort, countries of birth were aggregated into groups as a proxy. France was taken as the reference. The four other groups were “Europe + North America + Latin America + Australia,” “North Africa + Middle East,” “Sub-Saharan Africa + the Caribbean,” and “Asia.” Living in poverty was defined as a standard of living lower than the 2015 French poverty threshold (1 015€ per month) (Institut National de la Statistique et des Etudes Economiques 2021). Standard of living was calculated as the disposable income divided by the number of consumption units in the household. Educational level was dichotomized into having an upper secondary school certificate or not, and employment status as having a job or not.

Liver fibrosis was assessed using the FIB-4 index, a non-invasive marker of fibrosis calculated using age, AST level, ALT level, and platelet count with the following formula: age [years] * AST [IU/L])/(platelet count [10^9^/L] * (ALT [IU/L])^1/2^. Advanced liver fibrosis was defined as an FIB-4 index >3.25 (Sterling et al. 2006; Vallet-Pichard et al. 2007). History of HCV treatment was classified into “never” (treatment naive), “interferon-based” (with or without ribavirin), “1^st^ or 2^nd^ generation DAA” (i.e., telaprevir, ribavirine, asunaprevir, daclatasvir, boceprevir, ledispavir, simeprevir, sofosbuvir, dasabuvir, ombitasvir, paritaprevir, ritonavir, mericitabine, velpatasvir, grazoprevir, elbasvir, paritaprevir, glecaprevir, and pibrentasvir), and “other” (e.g., ribavirin alone).

### Statistical analyses

Study sample characteristics were compared according to (i) central obesity status and (ii) the three-category BMI status variable (participants with obesity, those with overweight, and those without obesity or overweight). Characteristics of excluded patients because of missing data on cannabis use, waist circumference, or BMI were compared with those of included patients. The Chi-squared and Student’s *t* tests were used in these comparisons for categorical and continuous variables, respectively.

Two separate analyses were performed to test the hypothesis that cannabis use is associated with lower corpulence. First, we estimated a binary logistic regression model with central obesity as the outcome. Second, we estimated a multinomial logistic regression model with the three-category BMI-status outcome (with “underweight or normal weight” as the reference). Associations were assessed by odds ratios (OR) for the logistic regression and by relative risk ratios (RRR) for the multinomial regression. Only variables with a liberal *p*-value < 0.20 in the univariable analyses were considered eligible for the multivariable models (Hosmer and Lemeshow 2000). Given the large number of explanatory variables and their potential multicollinearity, the final multivariable models were built using a backward stepwise selection procedure. This procedure removes one-by-one insignificant variables from the set of eligible variables based on the significance of their associations with the outcome. The likelihood ratio test (*p*< 0.05) was used to define the variables to keep in the final multivariable model. Subsequently, variables which were eligible for multivariable analyses but not retained in the final models (i.e., discarded during the backward stepwise procedure) were separately reintroduced in the latter to test for potential changes in terms of the level of significance of the associations and of changes in OR or RRR estimates, with a change-in-estimates threshold of 0.05 (Dunkler et al. 2014). Based on the same selection procedure and using linear regression models, we also tested whether cannabis use was associated with waist circumference and BMI as continuous variables.

To test for potential bias arising from the exclusion of participants with missing waist circumference measures, we performed a sensitivity analysis by including them in the analysis with the three-category BMI-status outcome, and compared the results with those of the main analysis.

All analyses were performed with Stata software version 16.1 for Windows (StataCorp LP, College Station, TX).

## Results

### Study population characteristics

**Fig. 1:**
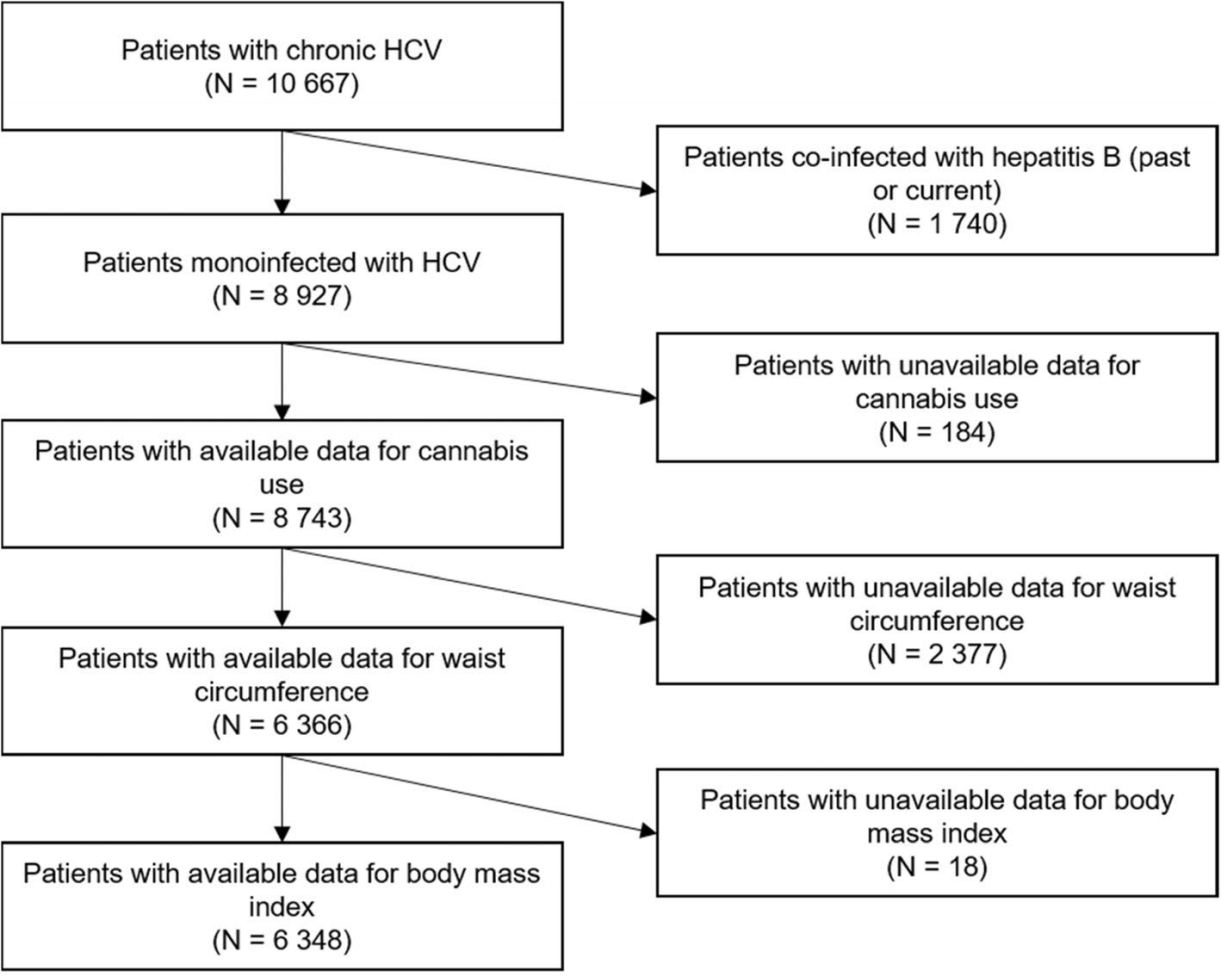
Flow chart of the study population (ANRS CO22 Hepather cohort)

The study population comprised 6348 participants (Fig. 1) whose characteristics are presented in Table 1. They were mainly male (53.8%), with a median age of 56 years (interquartile range [50-64]). Fifty-five percent had central obesity and 13.7% were obese according to their BMI. Thirteen percent had obesity according to both definitions. Current cannabis users represented 12.4% of the study population.

**Table 1:** Study population characteristics according to obesity status (ANRS CO22 Hepather cohort, *N* = 6348)

Variable (% of missing values)		Central obesity^a^			Underweight or normal weight	Overweight	Obesity	
	Study population (***N*** = 6348)	No (***N*** = 2832)	Yes (***N*** = 3516)	***P***-value^**b**^	BMI < 25 kg/m^**2**^ (***N*** = 3443)	25 ≤ BMI < 30 kg/m^**2**^ (***N*** = 2038)	BMI ≥ 30 kg/m^**2**^ (***N*** = 867)	***P***-value
	***N*** (%)	***N*** (%)	***N*** (%)		***N*** (%)	***N*** (%)	***N*** (%)	
**Sex**								
Male	3418 (53.8)	1744 (61.6)	1674 (47.6)	< 10^−3^	1708 (49.6)	1288 (63.2)	422 (48.7)	< 10^−3^
Female	2930 (46.2)	1088 (38.4)	1842 (52.4)		1735 (50.4)	750 (36.8)	445 (51.3)	
**Age at baseline (years)**								
Median [IQR]	56 [50–64]	53 [48–61]	58 [51–66]	< 10^−3^	55 [49–63]	57 [50–65]	57 [50–65]	< 10^−3^
**Place of birth (0.1)**								
France	4673 (73.6)	2188 (77.3)	2485 (70.7)	< 10^−3^	2671 (77.6)	1460 (71.7)	542 (62.6)	< 10^−3^
Europe + North America + Latin America + Australia^c^	486 (7.7)	221 (7.8)	265 (7.5)		246 (7.1)	160 (7.9)	80 (9.2)	
North Africa + Middle East	576 (9.1)	212 (7.5)	364 (10.4)		259 (7.5)	207 (10.2)	110 (12.7)	
Sub-Saharan Africa + the Caribbean^d^	412 (6.5)	117 (4.1)	295 (8.4)		153 (4.4)	157 (7.7)	102 (11.8)	
Asia	198 (3.1)	93 (3.3)	105 (3.0)		113 (3.3)	53 (2.6)	32 (3.7)	
**Coffee consumption (0.7)**								
0 cups/day	1795 (28.5)	733 (26.1)	1062 (30.4)	< 10^−3^	915 (26.8)	594 (29.3)	286 (33.3)	0.002
1–2 cups/day	2531 (40.2)	1087 (38.7)	1444 (41.4)		1 391 (40.7)	806 (39.8)	334 (38.9)	
≥ 3 cups/day	1977 (31.4)	991 (35.3)	986 (28.2)		1112 (32.5)	627 (30.9)	238 (27.7)	
**Cannabis use**								
Never	4271 (67.3)	1632 (57.6)	2639 (75.1)	< 10^−3^	2131 (61.9)	1446 (71.0)	694 (80.0)	< 10^−3^
Former	1292 (20.4)	680 (24.0)	612 (17.4)		753 (21.9)	410 (20.1)	129 (14.9)	
Current	785 (12.4)	520 (18.4)	265 (7.5)		559 (16.2)	182 (8.9)	44 (5.1)	
**Tobacco smoking**								
Never	2390 (37.7)	861 (30.4)	1529 (43.5)	< 10^−3^	1200 (34.9)	795 (39.0)	395 (45.6)	< 10^−3^
Former	1747 (27.5)	730 (25.8)	1017 (28.9)		866 (25.2)	616 (30.2)	265 (30.6)	
Current	2210 (34.8)	1241 (43.8)	969 (27.6)		1377 (40.0)	627 (30.8)	206 (23.8)	
**Alcohol consumption (0.4)**								
Abstinent without past unhealthy use	2723 (43.1)	1084 (38.4)	1639 (46.8)	< 10^−3^	1401 (40.8)	888 (43.8)	434 (50.4)	< 10^−3^
Moderate use	2521 (39.9)	1241 (44.0)	1280 (36.6)		1448 (42.2)	786 (38.7)	287 (33.3)	
Unhealthy use (past or current)	1077 (17.0)	496 (17.6)	581 (16.6)		582 (17.0)	355 (17.5)	140 (16.3)	
**Living in poverty (2.6)**								
No	4303 (69.6)	1974 (71.6)	2329 (67.9)	0.002	2411 (72.0)	1372 (68.8)	520 (61.7)	< 10^−3^
Yes	1883 (30.4)	782 (28.4)	1101 (32.1)		939 (28.0)	621 (31.2)	323 (38.3)	
**Education level (1.1)**								
< upper secondary school certificate	3396 (54.1)	1359 (48.5)	2037 (58.6)	< 10^−3^	1710 (50.3)	1148 (56.7)	538 (63.0)	< 10^−3^
≥ upper secondary school certificate	2883 (45.9)	1443 (51.5)	1440 (41.4)		1692 (49.7)	875 (43.3)	316 (37.0)	
**Employed (0.4)**								
No	3518 (55.6)	1308 (46.4)	2210 (63.1)	< 10^−3^	1797 (52.4)	1146 (56.4)	575 (66.7)	< 10^−3^
Yes	2807 (44.4)	1513 (53.6)	1294 (36.9)		1633 (47.6)	887 (43.6)	287 (33.3)	
**Advanced liver fibrosis**^e^ **(6.6)**								
No	4391 (74.0)	2049 (78.4)	2342 (70.6)	< 10^−3^	2429 (75.9)	1387 (72.3)	575 (70.8)	0.001
Yes	1541 (26.0)	566 (21.6)	975 (29.4)		772 (24.1)	532 (27.7)	237 (29.2)	
**Time since HCV diagnosis (years) (2.0)**								
Median [IQR]	14.0 [6.7–19.8]	14.4 [6.8–19.9]	13.7 [6.7–19.7]	0.126	14.4 [7.2–20.0]	13.8 [6.8–19.6]	12.7 [6.0–19.1]	0.002
**HCV treatment history**								
Never	3289 (51.8)	1586 (56.0)	1703 (48.4)	< 10^−3^	1938 (56.3)	960 (47.1)	391 (45.1)	< 10^−3^
Interferon-based	2278 (35.9)	947 (33.4)	1331 (37.9)		1146 (33.3)	794 (39.0)	338 (39.0)	
1^st^ or 2^nd^ generation DAA	616 (9.7)	226 (8.0)	390 (11.1)		267 (7.8)	231 (11.3)	118 (13.6)	
Other	165 (2.6)	73 (2.6)	92 (2.6)		92 (2.7)	53 (2.6)	20 (2.3)	

*BMI* body mass index, *DAA* direct-acting antiviral, *HCV* hepatitis C virus, *IQR* interquartile range

^a^Central obesity was defined as having a waist circumference ≥ 94 cm for men (except for men born in Asia, Central or South America, for whom the cut-off was set at 90 cm) and ≥ 80 cm for women (World Health Organization 2019)

^b^The chi-squared and Student’s *t* tests were used in these comparisons for categorical and continuous variables, respectively

^c^23 participants were born in Latin America, 15 in the USA, and 2 in Australia

^d^10 participants were born in the Caribbean

^e^Advanced liver fibrosis was defined as an FIB-4 score >3.25 (Sterling et al. 2006)

Excluded cohort patients differed from those included in the present study in terms of sex, place of birth, cannabis use (15.8% of excluded participants were current users), alcohol consumption, living in poverty, employment status, and time since HCV diagnosis (data not shown).

### Factors associated with central obesity

In the multivariable analysis, former (adjusted OR (aOR) [95% confidence interval (CI)]: 0.75 [0.64–0.88], *p*<0.001) and current (aOR [95% CI]: 0.45 [0.37–0.55], *p*<0.001) cannabis use were associated with a lower risk of central obesity (Table 2). Other protective factors included male sex, younger age, current tobacco use, having an upper secondary school certificate, and having a job. Conversely, risk factors included being born in “North Africa + Middle East” or in “Sub-Saharan Africa + the Caribbean,” unhealthy alcohol use (past or current), living in poverty, having advanced liver fibrosis, and previous HCV treatment with interferon or 1^st^ or 2^nd^ generation DAA (Table 2).

**Table 2:** Factors associated with central obesity in univariable and multivariable analyses (logistic regression, ANRS CO22 Hepather cohort, *N* = 6348)

Variables	Univariable analysis (***N*** = 6348)		Multivariable analysis (***N*** = 5742)	
	OR [95% CI]	***P***-value	aOR [95% CI]	***P***-value
**Sex**				
Male (ref.)	1		1	
Female	1.76 [1.60–1.95]	< 10^−3^	1.49 [1.31–1.67]	< 10^−3^
**Age at baseline (years)**	1.04 [1.03–1.04]	< 10^−3^	1.02 [1.01–1.02]	< 10^−3^
**Place of birth**		**< 10**^−**3**^		**< 10**^−**3**^
France (ref.)	1		1	
Europe + North America + Latin America + Australia	1.06 [0.88–1.27]	0.571	1.03 [0.83–1.27]	0.804
North Africa + Middle East	1.51 [1.26–1.81]	< 10^−3^	1.28 [1.04–1.57]	0.022
Sub-Saharan Africa + the Caribbean	2.22 [1.78–2.77]	< 10^−3^	1.90 [1.48–2.44]	< 10^−3^
Asia	0.99 [0.75–1.32]	0.967	1.02 [0.74–1.42]	0.890
**Coffee consumption**		**< 10**^−**3**^		
0 cups/day (ref.)	1			
1–2 cups/day	0.92 [0.81–1.04]	0.166		
≥ 3 cups/day	0.69 [0.60–0.78]	< 10^−3^		
**Cannabis use**		**< 10**^−**3**^		**< 10**^−**3**^
Never (ref.)	1		1	
Former	0.56 [0.49–0.63]	< 10^−3^	0.75 [0.64–0.88]	< 10^−3^
Current	0.32 [0.27–0.37]	< 10^−3^	0.45 [0.37–0.55]	< 10^−3^
**Tobacco smoking**		**< 10**^−**3**^		**0.001**
Never (ref.)	1		1	
Former	0.78 [0.69–0.89]	< 10^−3^	1.08 [0.93–1.26]	0.327
Current	0.44 [0.39–0.49]	< 10^−3^	0.81 [0.69–0.95]	0.009
**Alcohol consumption**		**< 10**^−**3**^		**0.002**
Abstinent without past unhealthy use (ref.)	1		1	
Moderate use	0.68 [0.61–0.76]	< 10^−3^	1.04 [0.91–1.18]	0.583
Unhealthy use (past or current)	0.77 [0.67–0.89]	< 10^−3^	1.35 [1.13–1.61]	0.001
**Living in poverty**				
No (ref.)	1		1	
Yes	1.19 [1.07–1.33]	0.002	1.18 [1.03–1.35]	0.019
**Education level**				
< upper secondary school certificate (ref.)	1		1	
≥ upper secondary school certificate	0.67 [0.60–0.74]	< 10^−3^	0.71 [0.63–0.80]	< 10^−3^
**Employment status**				
No (ref.)	1		1	
Yes	0.51 [0.46–0.56]	< 10^−3^	0.78 [0.69–0.89]	< 10^−3^
**Advanced liver fibrosis**^a^				
No (ref.)	1		1	
Yes	1.51 [1.34–1.70]	< 10^−3^	1.15 [1.00–1.31]	0.044
**Time since HCV diagnosis (years)**	1.00 [0.99–1.00]	0.250		
**HCV treatment history**		**< 10**^**−3**^		**0.001**
None (ref)	1		1	
Interferon-based	1.31 [1.18–1.46]	< 10^−3^	1.15 [1.02–1.30]	0.026
1^st^ or 2^nd^ generation DAA	1.61 [1.35–1.92]	< 10^−3^	1.49 [1.23–1.82]	< 10^−3^
Other	1.17 [0.86–1.61]	0.319	0.98 [0.68–1.42]	0.931

*aOR* adjusted odds ratio, *CI* confidence interval, *DAA* direct-acting antivirals, *OR* odds ratio, *ref.* reference group, *HCV* hepatitis C virus

^a^Advanced liver fibrosis was defined as an FIB-4 score >3.25 (Sterling et al. 2006)

Former and current cannabis use were also associated with a lower waist circumference after multivariable adjustment (linear regression coefficient (coef.) [95% CI]: −2.00 [−3.01; −1.00], *p*<0.001, and −5.43 [−6.66; −4.20], *p*<0.001, respectively) (data not shown).

### Factors associated with overweight and obesity as measured by BMI

In the multivariable analysis, both former and current cannabis uses were associated with a lower risk of both overweight and obesity (Table 3). Specifically, current cannabis use was associated with a 53% and 73% lower risk of overweight and obesity, respectively (*p*<0.001), *versus* no lifetime cannabis use. Former cannabis use was associated with a 22% and 42% lower risk of overweight and obesity (*p*=0.003 and *p*<0.001, respectively), *versus* no lifetime use.

**Table 3:** Factors associated with overweight and obesity in multivariable analyses (multinomial logistic regression, ANRS CO22 Hepather cohort, *N* = 6049)

Variables	Overweight (25 ≤ BMI < 30 kg/m^**2**^)		Obesity (BMI ≥ 30 kg/m^**2**^)	
	aRRR [95% CI]	***P***-value	aRRR [95% CI]	***P***-value
**Sex**				
Male (ref.)	1		1	
Female	0.46 [0.41–0.53]	< 10^−3^	0.86 [0.73–1.03]	0.094
**Age at baseline (years)**	1.01 [1.00–1.02]	0.002	0.99 [0.98–1.00]	0.062
**Place of birth**		**< 10**^−**3**^		**< 10**^−**3**^
France (ref.)	1		1	
Europe + North America + Latin America + Australia	1.10 [0.88–1.37]	0.397	1.39 [1.04–1.84]	0.024
North Africa + Middle East	1.20 [0.97–1.48]	0.093	1.67 [1.28–2.18]	< 10^−3^
Sub-Saharan Africa + the Caribbean	1.75 [1.35–2.28]	< 10^−3^	2.61 [1.93–3.53]	< 10^−3^
Asia	0.76 [0.53–1.08]	0.122	1.14 [0.74–1.75]	0.550
**Cannabis use**		**< 10**^−**3**^		**< 10**^−**3**^
Never (ref.)	1		1	
Former	0.78 [0.66–0.92]	0.003	0.58 [0.46–0.74]	< 10^−3^
Current	0.47 [0.38–0.59]	< 10^−3^	0.27 [0.19–0.39]	< 10^−3^
**Tobacco smoking**		**0.004**		**< 10**^−**3**^
Never (ref.)	1		1	
Former	1.02 [0.87–1.20]	0.773	1.38 [1.12–1.69]	0.002
Current	0.79 [0.66–0.94]	0.007	0.82 [0.65–1.03]	0.093
**Living in poverty**				
No (ref.)	1		1	
Yes	1.21 [1.05–1.40]	0.008	1.34 [1.11–1.62]	0.003
**Education level**				
< upper secondary school certificate (ref.)	1		1	
> upper secondary school certificate	0.81 [0.72–0.92]	0.001	0.61 [0.52–0.73]	< 10^−3^
**Employment status**				
No (ref.)	1		1	
Yes	1.02 [0.89–1.17]	0.769	0.69 [0.57–0.83]	< 10^−3^
**Time since HCV diagnosis (years)**	0.99 [0.98–1.00]	0.033	0.99 [0.98–1.00]	0.052
**HCV treatment history**		**< 10**^−**3**^		**< 10**^−**3**^
None (ref.)	1		1	
Interferon-based	1.33 [1.16–1.51]	< 10^−3^	1.46 [1.22–1.74]	< 10^−3^
1^st^ or 2^nd^ generation DAA	1.60 [1.30–1.96]	< 10^−3^	2.13 [1.64–2.76]	< 10^−3^
Other	1.20 [0.83–1.72]	0.338	1.02 [0.60–1.73]	0.948

*aRRR* adjusted relative risk ratio, *BMI* body mass index, *CI* confidence interval, *DAA* direct-acting antivirals, *ref.* reference group, *HCV* hepatitis C virus

Former and current cannabis use were also associated with a lower BMI level after multivariable adjustment (coef. [95% CI]: −0.77 [−1.07; −0.47], *p*<0.001, and −1.88, [−2.25; −1.51], *p*<0.001, respectively) (data not shown).

The following variables were associated with both definitions of obesity in multivariable analyses (Tables 2 and 3): cannabis use, living in poverty, education level, employment status, being born in “North Africa and Middle East” or “Sub-Saharan Africa,” and history of HCV treatment.

None of the reintroductions of excluded explanatory variables in the final models had a relevant impact on the model estimates. Sensitivity analyses, performed on the population that included participants with no data on waist circumference, led to similar results to those found in the main analysis in terms of significance level and adjusted RRR magnitude (data not shown).

## Discussion

Using cross-sectional data from 6348 chronically infected HCV patients, we found that current cannabis use was associated with a 55% lower likelihood of central obesity (elevated waist circumference), a 73% lower likelihood of obesity (BMI ≥ 30 kg/m^2^), and a 53% lower likelihood of overweight (BMI between 25 and 30 kg/m^2^), when compared with no lifetime cannabis use. To our knowledge, this is the first time that such associations have been highlighted for HCV-infected patients.

Our results are in line with those found in general populations in different countries (Meier et al. 2019; Alshaarawy and Anthony 2019; Sidney 2016; Ngueta et al. 2015; Clark et al. 2018). For instance, Ngueta et al., among Inuit adults, found an OR of 0.56 for obesity (BMI ≥ 30 kg/m^2^) for past-year cannabis use as compared to non-users (Ngueta et al. 2015). They also found a similar association between frequent cannabis use (but not former nor infrequent use) and concurrent elevated triglycerides and waist circumference (Ngueta 2020). However, causal association was not found in a recent Mendelian randomization study (Alayash et al. 2021). Our results are also in line with what we found in a previous study for patients with chronic hepatitis B virus from the same cohort (Barré et al. 2021a).

One possible explanation for the relationship between cannabis use and corpulence is that such frequent use may downregulate cannabinoid receptor 1 (CB1)—which regulates appetite and body weight, thereby reducing energy storage and increasing metabolic rates (Clark et al. 2018; Spindle et al. 2021). In two preclinical studies, CB1 antagonists and peripherally restricted CB1 antagonists (i.e., with no effect on the central nervous system) showed some efficacy on obesity and metabolic syndrome (O’Sullivan et al. 2021; Lopez Trinidad et al. 2021).

However, assuming that the effects of cannabis are solely attributable to the most abundant phytocannabinoid Δ9-tetrahydrocannabinol (THC) (Cluny et al. 2015) and/or are only mediated through CB1 may be reductive. Cannabis exposes users to a large number of phytocannabinoids, as well as to non-cannabinoid molecules, such as terpenoids (Russo 2018). The most abundant phytocannabinoids in cannabis are THC, cannabidiol (CBD), and Δ9-tetrahydrocannabivarin (THCV). Beside their varying affinity for CB1 and CB2, these cannabinoids also interact with other targets such as transient receptor potential channels, GPR55 receptor, or peroxisome proliferator activated receptors, which in turn may impact energy metabolism (Bielawiec et al. 2020; Abioye et al. 2020). Moreover, cannabis compounds may have anti-oxidant and anti-inflammatory properties (Atalay et al. 2019; Bielawiec et al. 2021; Henshaw et al. 2021; Graczyk et al. 2021), inflammation being a major element in chronic diseases, especially metabolic and obesity-related disorders (Cavalheiro et al. 2022; Ellulu et al. 2017). Non-cannabinoid cannabis compounds such as limonene, β-caryophyllene, and other terpenes may also play a role in the corpulence lowering effect of cannabis use (Hashiesh et al. 2021; Jing et al. 2013; Scandiffio et al. 2020). Finally, a synergetic effect, resulting from the interactions between those compounds, cannot be excluded (Russo 2018).

By lowering body weight, cannabis use may have an indirect beneficial effect on liver disease and metabolic disorders in patients with chronic HCV infection. Cannabis and/or cannabis compounds may also have a direct beneficial effect on these problems. More specifically, a growing body of research, including observational, preclinical, and clinical data, suggests that phytocannabinoids may play a role in the prevention or treatment of hepatic steatosis (Berk et al. 2021; Barré et al. 2021b). As modulators of the endocannabinoid system, which is a main therapeutic target for treating diabetes mellitus (Veilleux et al. 2019), cannabis compounds may exert a beneficial role on the development of diabetes in different populations (Bielawiec et al. 2020; Wargent et al. 2013; Jadoon et al. 2016; Meah et al. 2021), including HCV-infected people (Barré et al. 2020). Observational studies have also highlighted a potential role of cannabis compounds in liver disease prevention in this population (Adejumo et al. 2018; Santos et al. 2020).

We adjusted our analyses for tobacco use, which is common in European cannabis users (Hindocha et al. 2016) and is associated with lower body weight (Audrain-McGovern and Benowitz 2011). Accordingly, the effect of cannabis use we found cannot be imputed to tobacco use.

The other factors associated with both definitions of obesity in our study were socioeconomic status, African origin, and history of HCV treatment. The inverse relationship between socioeconomic status and elevated body weight/obesity is multifactorial and has been widely documented (Newton et al. 2017; Vieira et al. 2019; Wang and Beydoun 2007; Pigeyre et al. 2016; Hruby et al. 2016). A higher BMI and/or risk of obesity in African-Americans, as well as immigrants from Africa in Europe, has also been reported (Shai et al. 2006; Agyemang et al. 2016; Toselli et al. 2014; Min et al. 2021; Abraham et al. 2013). This is a complex phenomenon and involves socio-cultural, economic, and genetic factors.

We found that a history of HCV treatment (interferon-based and 1^st^/2^nd^ generation DAA) was associated with a higher risk of obesity. This may seem counter-intuitive given the documented weight loss-inducing effect of interferon (Alam et al. 2013). Elsewhere, elevated BMI/obesity was an independent risk factor for nonresponse to previous antiviral treatment (Bressler et al. 2003; Asselah et al. 2010). Therefore, as HCV-cured patients were not included in our analyses, participants with a history of HCV treatment were more likely to have risk factors for nonresponses (i.e., obesity) than those with no such history.

The main strength of the present study is its large sample size. The inclusion of socio-behavioral factors is another strength, as it is widely recognized that these factors greatly impact body weight. The assessment of corpulence through two distinct markers (waist circumference and BMI) and the consistency of our results across the different models suggest they are robust. It has previously been highlighted that a measure of waist circumference provides both independent and complementary information to the BMI value when predicting morbidity and mortality. This is likely to be at least partly due to its ability to identify adults with increased visceral adipose tissue mass (Ross et al. 2020).

Some study limitations need to be acknowledged. First, cannabis use was self-declared, and we therefore cannot exclude potential desirability bias, which was however equally likely in people with and without obesity. Second, data on frequency of cannabis use were not collected, preventing the possibility of conducting dose-response analyses. Similarly, cannabinoid content of cannabis products was unknown. However, most recent data indicated that THC levels are increasing in both herb and resin in France and other European countries, reaching around 11 and 18% THC in France, respectively (Freeman et al. 2021; Gandilhon et al. 2019). Third, data for some of the most important determinants of elevated body weight (i.e., dietary intakes and physical activity) were not available. However, we can assume that socioeconomic variables captured part of these effects.

## Conclusions

To conclude, we found that both former and, to a greater extent, current cannabis use were consistently associated with lower waist circumference, lower BMI values, and lower risks of overweight, obesity, and central obesity in patients with chronic HCV infection. Longitudinal studies are needed to confirm those relationships and assess the effect of cannabis use on corpulence and on liver outcomes after HCV cure.

## Data Availability

The datasets generated and/or analyzed during the current study are not publicly available due to ongoing data treatment; however, they are available from the corresponding author upon reasonable request.

## Abbreviations

ALT: Alanine aminotransferase
aOR: Adjusted odds ratio
aRRR: Adjusted relative risk ratio
AST: Aspartate aminotransferase
BMI: Body mass index
CB1: Cannabinoid receptor 1
CBD: Cannabidiol
DAA: Direct-acting antiviral
HCV: Hepatitis C virus
HBV: Hepatitis B virus
IQR: Interquartile range
THC: Δ9-Tetrahydrocannabinol
THCV: Δ9-Tetrahydrocannabivarin
